# Feasibility of preoperative tattooing of percutaneously biopsied axillary lymph node: an experimental pilot study

**DOI:** 10.1186/s40814-020-00682-2

**Published:** 2020-09-24

**Authors:** Abida K. Sattar, Basim Ali, Imrana Masroor, Shaista Afzal, Mohammad Usman Tariq, Romana Idrees, Maseeh Uzzaman, Wardah Khalid

**Affiliations:** 1 Department of Surgery, Aga Khan University, Link Building, Stadium Road, Karachi, 74800 Pakistan; 2 Department of Biological and Biomedical Sciences, Aga Khan University, Karachi, Pakistan; 3 Department of Radiology, Aga Khan University, Karachi, Pakistan; 4 Department of Pathology, Aga Khan University, Karachi, Pakistan

**Keywords:** Axillary lymph node dissection, Sentinel lymph node biopsy, Neoadjuvant chemotherapy, Preoperative tattooing of the biopsied lymph node, Black or India ink

## Abstract

**Background:**

In the last three decades, axillary lymph node dissection (ALND) has been replaced by sentinel lymph node biopsy (SLNB) in all clinically node-negative patients. However, when SLNB alone is performed in clinically node-positive patients who are rendered node-negative by neoadjuvant chemotherapy, the procedure has a high false-negative rate and other complementary procedures have been described to improve its reliability. Preoperative tattooing of the suspicious lymph node with India ink at the time of biopsy, in addition to sentinel lymph node biopsy, is a reasonable alternative. The objective of our study is to determine, in clinically node-positive patients, the feasibility of tattooing suspicious axillary lymph node at the time of percutaneous needle biopsy and its retrieval at the time of surgery.

**Methods:**

A prospective experimental study will be conducted divided into two phases—phases I and II. In phase I, 10 patients committed to undergo upfront surgery (without neoadjuvant chemotherapy) will have a suspicious lymph node tattooed by injecting India ink at the time of core needle biopsy. All patients will undergo a SLNB, during which the axilla will be inspected to determine if the tattooed lymph node can be visualized. Routine microscopic examination will follow, and concordance between the sentinel and tattooed node will also be established. In phase II, the process will be repeated for 30 patients who undergo surgery after neoadjuvant chemotherapy. The analysis will be performed in Stata version 12.

**Discussion:**

There is a need to identify and test the techniques for the down-staged axilla in post-neoadjuvant chemotherapy patients, which are not only practical and limit the number of invasive procedures necessary but are representative of the new axillary status and help limit the extent of axillary surgery without negatively impacting outcomes. We propose that, for the patient undergoing neoadjuvant chemotherapy with a biopsy-proven disease in the axilla, this could be achieved by India ink which allows marking, identification, and retrieval of the biopsied lymph node. Retrieval of this previously biopsied lymph node along with sentinel nodes, if found to be representative of the status of the remainder of the axilla, could potentially eliminate the need for routine axillary lymph node dissection and thus limit morbidity.

**Trial Registration:**

ClinicalTrials.gov, NCT03939598. Retrospectively registered on 7 May 2019.

## Background

Breast cancer is the most common cancer in females causing over 9.6 million cancer deaths globally in 2018 [1]. The current mainstay of treatment of non-stage IV breast cancer employs a multidisciplinary approach using surgery, often in combination with radiation and systemic therapy. The sequence of the therapies given is not only dictated by the clinical stage of the disease but also by the tumor biology. There is growing evidence suggesting the survival benefit of NAC in addition to its ability to down-stage disease and improve operability [2, 3]. Axillary nodal evaluation remains critical in breast cancer care, as its status is a strong predictor of long term survival and recurrence.

Management of the axilla in early breast cancer historically required complete axillary lymph node dissection (ALND) [4]. However, there was associated morbidity and decreased quality of life due to complications of lymphedema, chronic pain, and decreased range of motion. In the last three decades, ALND has been replaced by sentinel lymph node biopsy (SLNB) in all clinically node-negative patients, regardless of the tumor size or its biology. The success of SLNB has helped avoid routine ALND in up to 60% of such patients with equivalent survival and lower morbidity than those undergoing ALND [5, 6]. In patients who are clinically node-negative and receive NAC, SLNB is performed at the time of definitive surgery after NAC with similar success.

However, when node-positive patients undergo NAC, the management of the axilla is not as straightforward. The use of SLNB alone even after downstaging the axilla has a high failure rate [7, 8]. Currently, there is a lack of consensus on a technique that can be used to accurately predict the status of the axilla in such patients while avoiding the morbidity of a routine ALND [9].

In the clinically node-positive patient undergoing NAC, as SLNB alone is considered suboptimal, other techniques (such as the TAD and MARI procedures) aimed at reducing the false-negative rate as well as limiting the extent of axillary surgery have been described [10, 11]. All such techniques warrant percutaneous biopsy and marking of the biopsied lymph node prior to initiating NAC. They also require an additional invasive procedure performed preoperatively to localize this previously marked lymph node to facilitate its retrieval intraoperatively. Retrieval of the biopsied/marked lymph node in addition to the sentinel nodes remains critical as it has been shown to lower the false-negative rate.

Preoperative tattooing of the suspicious lymph node with India ink at the time of biopsy is a reasonable alternative as it would mark the suspicious lymph node and help its retrieval. Tattooing of axillary lymph nodes has been described previously but whether or not it has a success rate comparable to clipping the lymph node in staging the axilla after NAC is unclear [12].

We hypothesize that using only one invasive procedure preoperatively, black India ink could be used to mark, identify, and retrieve the previously biopsied lymph node at the time of surgery. In the patients who have received NAC, this technique coupled with SLNB may help avoid routine ALND.

The objective of our study is to determine, in clinically node-positive patients, the feasibility of tattooing suspicious axillary lymph node at the time of percutaneous needle biopsy and its retrieval at the time of surgery.

## Outcomes and feasibility hypothesis

Our study is divided into two phases, and progress from phase I to II will be dependent on the success of the first (see the “Study design” section).

To evaluate the feasibility of using India ink to tattoo and retrieve axillary lymph nodes, we will study three main outcomes:

1. Rate of intraoperative identification of tattooed lymph node in:
  - Upfront surgery group (phase I)
  - Neoadjuvant group (phase II)
2. Concordance of the tattooed lymph node with sentinel lymph node (phases I and II)
3. False-negative rate of tattooing suspicious lymph nodes as assessed by a backup ALND (phase II)

## Methods

A prospective experimental pilot study will be conducted at the Aga Khan University to determine the feasibility of tattooing clinically suspicious axillary lymph nodes. Ethical approval was obtained from the Aga Khan University Hospital Ethics Review Committee (ERC). Patients will be enrolled from March 27, 2019, to December 31, 2020. The study was registered with Clinicaltrials.gov.

### Study design

**Fig. 1:**
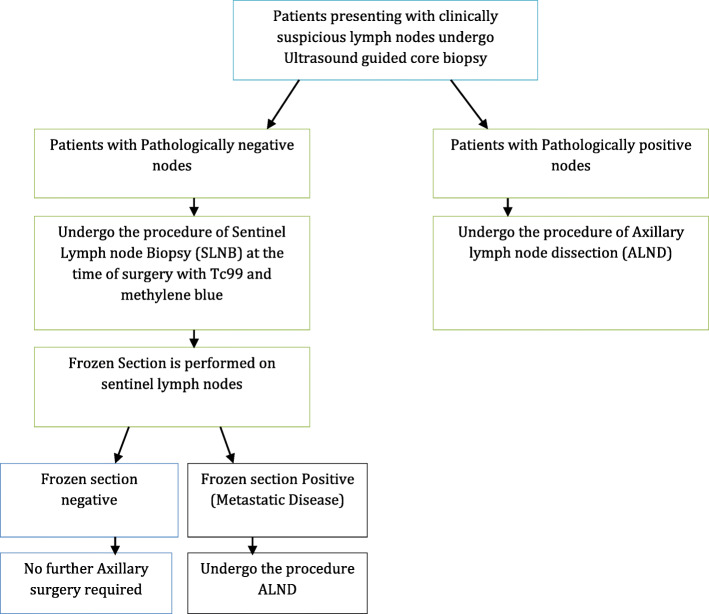
Current standard of management protocol for patients with clinically suspicious lymph nodes at Aga Khan University

The study will be carried out in two phases—phase I and phase II. Patients who have a strong clinical suspicion or biopsy-proven breast cancer and are noted to have suspicious axillary lymph nodes on clinical exam or axillary ultrasound will be considered for the study. Such patients will then undergo an ultrasound-guided core needle biopsy of the breast (if not already done) as well as of the suspicious axillary lymph node as per our current institutional protocol (Fig. 1). The suspicious/biopsied lymph node will also be tattooed with India ink at the time of biopsy.

Phase I will be conducted first and will include 10 patients who are planned to undergo upfront breast surgery without a need for NAC. This phase of the study will help to quickly evaluate (without having to wait 4–6 months for the course of NAC) if the axillary lymph node tattooing with India ink is feasible and if this tattooed lymph node can be identified intraoperatively and on pathological evaluation.

**Fig. 2:**
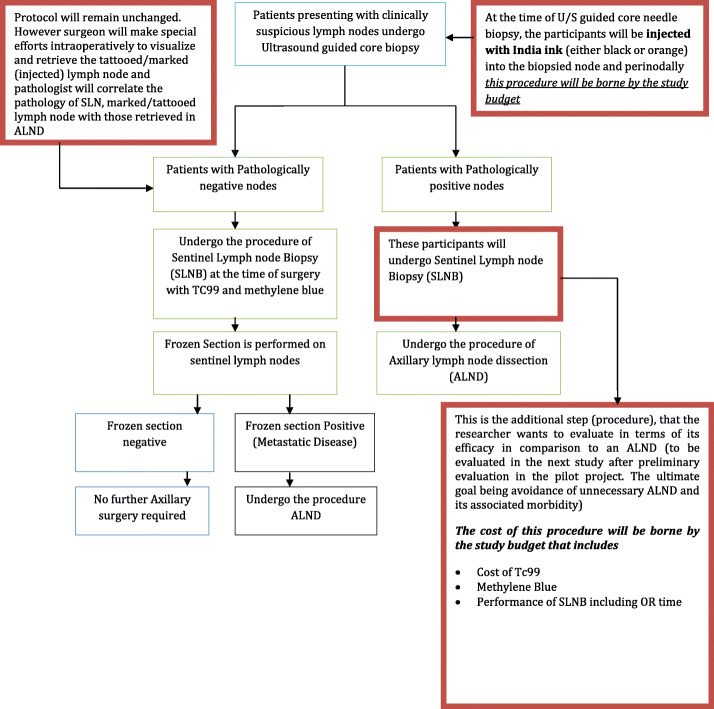
Study flow diagram: with additional steps for study in bold margins embedded with the current standard of management protocol for patients with clinically suspicious lymph nodes at Aga Khan University

Phase II will be conducted after phase I, if phase I is successful (based on the pre-set rate of intraoperative identification rate of ≥ 90%). In phase II, 30 patients who are planned to undergo neoadjuvant chemotherapy prior to definitive surgery will be included. This phase of the study will help evaluate if the tattooed lymph node can be identified intraoperatively and on pathological evaluation, even after a lag period of 4–6 months (while the patient received neoadjuvant chemotherapy) and also help determine its concordance with the sentinel lymph nodes. In those patients that had biopsy-proven evidence of disease in the lymph node and are committed to an ALND, performing the SLNB in addition to the planned ALND will help provide insight into the application of this technique in future trials (Fig. 2).

Based on a previous study evaluating lymph node tattooing, we hypothesize that both phases will be feasible if the tattooed nodes are visualized in ≥ 90% of our patients [12].

### Study procedure

Female patients with clinical suspicion of breast cancer, who meet the inclusion criteria (see below) will be approached to participate in the study. As per institutional guidelines, patients will undergo routine clinical breast examination by a specialist breast surgeon and appropriate radiological imaging.

Patients with clinically suspicious axillary lymph nodes will undergo an ultrasound-guided core needle biopsy of a single, most suspicious lymph node by study radiologists. In case multiple suspicious nodes are identified, the single most suspicious (see the “Inclusion criteria” section) and/or the largest suspicious/most easily accessible node will be tattooed. At the time of biopsy, immediately after obtaining the specimen, the biopsied lymph node will also be tattooed by the radiologist. For the purpose of tattooing, the patient will be positioned on the examination table in a lateral decubitus or supine position with the ipsilateral arm extended to 180° and placed by the side of the head. Back support will be provided to the patient. Core biopsy of the suspicious lymph node seen on ultrasound will be performed using an 18-gauge needle followed by tattooing of the lymph node using sterile, black India ink via a 25-gauge needle. India ink will be injected in the anterolateral cortex of the biopsied lymph node. The volume of tattoo ink will be determined based on the size of the suspicious lymph node: 0.3 ml of the tattoo ink will be injected if the size is between 3 and 5 mm and 0.5 ml if it is greater than 5 mm. India ink is sterilized on the day of intended use, in the pharmacy under a laminar hood by passing it through a 0.2-μm filter.

In all cases, whether pathologically negative or positive on biopsy, a SLNB will be performed at the time of definitive surgery, with dual tracer technique using technitium-99 (TC^99^) sulfur colloid and methylene blue as per current institutional protocol. For this purpose, after the injection of TC^99^ and lymphatic mapping preoperatively, diluted methylene blue will be injected into the subareolar plexus intraoperatively. The axillary hot spot will be assessed and documented for presence or absence, using a gamma probe. Sentinel lymph node biopsy will be performed in the usual fashion including identification of palpable lymph nodes. The total number of sentinel lymph nodes retrieved, number of hot, number of blue, and number of hot and blue will be documented.

Intraoperatively, the axilla will be inspected by the surgeon to determine the following: (a) whether the tattooed lymph node can be visualized and (b) whether the tattooed lymph node is easily distinguishable from methylene blue and is the tattooed lymph node also the SLNB.

Those patients with biopsy-proven axillary metastatic disease on core needle biopsy will proceed to have the planned breast procedure and axillary lymph node dissection. Management of the axilla in those participants who did not have biopsy-proven metastatic disease on axillary core needle biopsy (but had a suspicious node leading to core biopsy) will be dictated by the results of the frozen section analysis. If the frozen section results show the absence of metastatic disease in the submitted lymph nodes, no additional axillary surgery will be performed. While the frozen section is in process and results are awaited, the surgeon may proceed with the planned breast procedure.

In phase I of the study, patients with a negative biopsy of axillary lymph nodes will be included in the study as the purpose is to evaluate the visibility of the India ink tattoo and the ability to differentiate it from the sentinel node. In phase II, the patients found to have a negative biopsy of axillary lymph nodes will be excluded from the study.

In phase II, for patients who have received neoadjuvant chemotherapy, the extent of fibrosis (mild, moderate, or severe) will be documented. All submitted lymph nodes will be evaluated by two independent pathologists to ascertain the visualization of black India ink within the nodes and usual histologic assessment of all submitted specimens. Correlation between tattooed and sentinel nodes (including palpable) will be determined. Tumor pathology, including type, grade, receptors (ER, PR, and Her-2/neu), will be reported. A breast surgeon, radiologists, and pathologists will participate in the study. Additionally, the participating health professionals will be given similar training and instructions so as not to cause variability between those performing similar roles.

### Inclusion criteria

The following are the inclusion criteria:

- Female greater than 18 years of age of any BMI.
- Breast cancer patient (biopsy-proven or clinically suspected) with clinically suspicious (palpable on clinical exam or abnormal by ultrasound criteria but not biopsied yet) ipsilateral axillary lymph node(s). The ultrasonographic examination will be considered suspicious for metastasis if one or more of the following criteria are present: (1) eccentric cortical enlargement (> 3 mm) or lobulation with a displacement of the hilum, (2) absent hilum and irregular border and hypoechoic echotexture, (3) spherical node, and (4) perinodal vascularity [13–15].
- Participants willing to undergo axillary lymph node percutaneous biopsy with tattooing of the biopsied lymph node at Aga Khan University Hospital.
- Participants intending to have definitive surgery at Aga Khan University Hospital.

### Exclusion criteria

The following are the exclusion criteria:

- Participants with a terminal disease like renal failure will be excluded because these conditions can have a profound effect on their course of treatment.
- Participants with distant metastases.
- Participants with prior ipsilateral breast or axillary surgery.
- Participants with bilateral breast cancer.
- Participants who have already had an axillary core needle biopsy prior to inclusion (to avoid a second procedure for tattooing).
- Participants with recurrent breast malignancy because their course of treatment might be different.
- Pregnant and lactating women.
- Men with breast cancer.

### Study setting, sample size, and sampling technique

The study will be conducted at a single institution in Karachi, Pakistan—the Aga Khan University Hospital (AKUH), a tertiary care hospital in Karachi, Pakistan. The study participants will be recruited through a purposive sampling technique from consecutive patients presenting to the breast surgery clinic at AKUH. The sample size assumptions were based on the reported proportion of correctly identified tattooed lymph of 93% [12], with a confidence level of 95% and a design effect of 1; the minimum sample size calculated is 100 breast cancer patients. Since we will conduct a feasibility study, it will be conducted on 40% of the calculated sample size, i.e., on 40 breast cancer patients. The sample size was calculated through Open-EPI version 3.01.

### Statistical analysis

The analysis will be performed on Stata version 12. Descriptive analysis will be performed. For continuous variables, either the mean ± S.D or the median with IQR will be reported depending on the normality assumption of the variables. For categorical variables, frequency with percentages will be reported. The proportion of the lymph node visualized intraoperatively with black India Ink will be reported. Concordance between the sentinel lymph nodes and tattooed biopsied lymph nodes will be reported. False-negative rate (FNR) will be determined to assess the findings of preoperative pathological assessment against the final histological findings of the resected nodes, and 95% confidence interval (CI) for FNR will be calculated by Fisher’s exact test for the binomial proportions.

### Data collection and storage

Data collectors will be trained by the principal investigator (PI). All the forms used for the collection of data will be checked by PI before being keyed. The data will be double-entered by trained data entry operators. The PI will check for the accuracy of data entry, and discordant and/or missing information will be compared with original forms used for data collection. Data collectors will be reinforced to recruit only those participants who will be willing to participate in the study. The PI herself will be the surgeon performing the ALND and SLNB procedures and will have oversight of all major aspects of the study including data collection, entry, verification, and statistical analysis. Personnel involved in these roles will report directly to the PI. The team inclusive of the PI, radiologists, pathologists, data entry and collection staff, and statistician will meet every 4-6 months to review (a) the procedure notes (including those from biopsy, tattooing procedure, and surgery), (b) any challenges and unanticipated events encountered during the study, and (c) the data collected and analyzed until that point.

All study materials containing personal identifiers will be kept in a locked file cabinet. A unique study identification number will be assigned to each participant. After that, data will be entered from hard copy into the electronic database that will be password-protected and only accessed by the research staff of the study. The final dataset will be accessible to the PI, authors, and personnel involved in the data collection and storage. After the completion of the study, the dataset will remain with the PI.

### Safety and adverse events

Participants will be explained in detail regarding the risks associated with the procedure. Although sterile black India ink is safe to administer, possible side effects can include mucosal inflammation, due to spillage of the ink, abscess formation, and allergic reaction [16]. The responsibility of complications resulting from the additional steps of study procedures will be fully borne by the institution and study funds. Participants will be allowed to leave the study at any point in time, and they will continue to receive quality standard treatment services at AKUH. An office telephone number will be provided to address any patient concerns. Any adverse events will be documented by physicians and reported to the ClinicalTrials.gov and in the results of the study, when published.

## Discussion

For the purpose of axillary lymph node evaluation in the clinically node-positive patient where NAC may have down-staged axillary burden of disease, there is a need to identify and test the techniques that are practical, cost-effective, limit the number of invasive procedures, and are predictive of the status of the axilla. Such techniques should also be helpful in limiting the extent of axillary surgery and its associated morbidity without negatively impacting outcomes. We propose that for the patients undergoing neoadjuvant chemotherapy with clinically node-positive disease, this goal could be achieved by using India ink to preoperatively mark the suspicious lymph node at the time of needle biopsy. If this previously biopsied and tattooed lymph node is found to be representative of the status of the axilla in addition to the sentinel lymph nodes, the technique could potentially eliminate the need for routine ALND in this cohort of patients. The success of this pilot will have to be followed by a study with a greater sample size to confirm the efficacy of this procedure.

Using SLNB alone in the down-staged axilla has a high false-negative rate, which has warranted a need to refine the technique [7, 8]. Newer techniques described in the literature to mark and subsequently retrieve previously biopsied and marked lymph nodes along with the SLNB include procedures such as TAD and MARI [10, 11]. Most of these techniques described to retrieve the marked lymph node require an additional invasive procedure performed preoperatively, such as placement of a radioactive seed or a localization wire to facilitate intraoperative identification and retrieval.

ACOSOG Z1071 evaluated the efficacy of SLNB compared to ALND in node-positive patients who underwent NAC. It reported a false-negative rate of 12.6% with single-agent SLNB mapping and 10.8% for dual agent mapping (both higher than the pre-set acceptable rate of 10%). Additionally, the false-negative rate was dependent on the number of lymph nodes retrieved, with 3 nodes being the number that leads to an acceptable FNR of < 10% [7]. However, it is well known that the retrieval of 3 sentinel lymph nodes is not always guaranteed in SLNB.

In a subset of patients in ACOSOG Z1071, a clip was placed in the suspicious lymph node and retrieved at the time of biopsy which reduced the FNR to 6% [17]. A study by Caudle et al. showed that targeted dissection with the retrieval of clipped nodes and sentinel lymph nodes reduced the FNR to an impressive 2% [11].

Clip placement, however, comes with additional costs and issues of availability of Hydromark T3 polymer. Radioactive seed placement for localization is not widely available, expensive, and requires expertise and a separate invasive procedure. In addition, clips placed in the axilla are not always identifiable at the time of surgery and may be a source of future litigation [18]. The placement of clips to mark the biopsied lymph node has also lead to some difficulty in pathologic interpretation due to reaction to the clip which leads to a higher false-positive rate and potentially unnecessary axillary dissections [11, 17, 19].

Preoperative tattooing of the suspicious lymph node with India ink at the time of biopsy is a reasonable alternative as it would mark the clinically positive node. Since it has been shown to be intraoperatively visible, this technique would also avoid the additional procedure to facilitate intraoperative identification and retrieval and may avoid the false-positive interpretation as a reaction to the clip [12, 20]

Tattooing the lymph node is also a potentially simpler and cost-effective procedure. It has been successfully used in gastroenterology to mark areas of polypectomy and areas of resection. One recent study that evaluated the feasibility of the tattooing axillary lymph nodes with black ink before NAC identified that the black ink was visible in 93% of the patients intraoperatively and lasted for almost 7 months in cases where lymph node retrieval was performed after NAC [12].

Our study has several limitations as it is a pilot study. While the small number of patients enrolled will be appropriate for a pilot study, we will be unable to assess the efficacy of preoperatively tattooing lymph nodes to stage the axilla in patients receiving neoadjuvant chemotherapy. If this study is successful, we hope to pursue a larger, multi-center study evaluating the efficacy of this technique in predicting the status of the axilla, in conjunction with SLNB, to ultimately avoid routine axillary dissections in node-positive patients who are down-staged by the use of NAC.

The injection of India ink into the lymph node may have a learning curve. Additionally, some cultures/religions object to tattooing of the external skin, and we anticipate that some patients may object to tattooing internal lymph nodes. Phase I of our study will thus help to determine the acceptability of tattooing.

In conclusion, the success of this study will help to evaluate whether suspicious lymph nodes that are tattooed preoperatively are retrievable intraoperatively despite a lag while administering NAC. Whether the tattooed lymph node excised along with the sentinel node is representative of the status of the axilla may not be established definitively with the results of our study. However, the results of our study will aid in designing and implementing future larger studies to answer this question and limit the extent of axillary surgery.

## Data Availability

The full protocol is available publicly on ClinicalTrials.gov. The results, when available, will be shared with ClinicalTrials.gov and published.

## Appendix

**Table:**

**Project information**	
**Feasibility of preoperative tattooing of percutaneously biopsied axillary lymph node, its retrieval at surgery and correlation with sentinel lymph nodes in breast cancer patients: a pilot study**	Sponsor: Departmental of Surgery
ERC Ref No: 2018-0345-332	Organization: AKU
**Principal Investigator:** Abida K. Sattar	Location: Link Building
**Other investigators:** Basim Ali, Imrana Masroor, Shaista Afzal, Mohammad Usman Tariq, Romana Idrees Maseeh Uzzaman, Wardah Khalid	Location: Link Building
Phone: 02134864751	Organization: AKU

### Purpose of this research study

This study will be conducted by the Department of Surgery, Aga Khan University. We are from the Department of Surgery, AKUH. The purpose of this study is to find out whether sterile black = ink injected into the lymph node at the time of percutaneous lymph node biopsy is identifiable and retrievable at the time of surgery, regardless of whether surgery is performed either upfront or delayed, i.e., after neoadjuvant chemotherapy. This new technique is the first step and successful, it may ultimately lead to a second study, results of which may help us in limiting the extent of surgery on the axilla in a selected group of breast cancer patients. As one of the newly diagnosed breast cancer patients, you are a suitable person for this study. You are being asked to participate in the study as your listed age is greater than 18 years.

### Procedures

Before the start of treatment, a core needle biopsy of your axillary (armpit) lymph node will be performed under ultrasound guidance, which is a standard procedure at AKU at this time. For the experimental part of the study, the lymph biopsy will be immediately followed by an injection of sterile India ink into the biopsied lymph node to mark the lymph node which may help in retrieval of this particular node at the time of surgery as well as pathological after surgery. Though this India ink injection into the lymph node has not been done at AKU, this has been tried with success in the USA. This procedure will be performed by a trained radiologist in the Radiology Department at AKU. The procedure will take an additional 5 min. Once the results of your biopsy become available, you will meet with your surgeon for further management and a discussion regarding the timing of surgery and need for any medications such as chemotherapy prior to your surgery. At the time of surgery, your axilla will be evaluated initially by a sentinel lymph node biopsy which is a standard and well established surgical procedure. During this procedure, we will attempt to look for the marked/tattooed lymph node, which will also be retrieved and analyzed like all other lymph nodes. The need for additional surgery on the axilla will be determined based on the results of core needle biopsy as well as the testing of lymph nodes during surgery.

### Possible risks or discomfort

There are no major adverse effects associated with injecting of sterile black India ink inside the body as this is a routine practice in gastroenterology. But since this is the first time that we are performing this procedure in axillary lymph nodes, there might be chances of adverse reactions like swelling, itching, unintended permanent tattooing of skin at entry site or otherwise and allergic reaction. In case of any associated side effects due to India ink injection the treatment will be provided at AKUH and the cost will be bared by study budget.

### Possible benefits

The results of the study will be shared with you and your family. There will be no associated direct benefit to you. But if this technique is proven to be efficacious, it can be further applied to test the efficacy of SLNB against ALND in upcoming future studies, thus possibly limiting the extent of surgery on the axilla in the future and avoiding associated complications. Eventually, this will be beneficial for the individual patient and the scientific community in the future.

### Financial considerations

No financial consideration will be given.

### Available treatment alternatives

Treatment facilities available at AKU will be available for the participants according to the institutional guidelines.

### Available medical treatment for adverse experiences

Any adverse effects encounter due to the injection of sterile black India ink and methylene blue dye or due to any study procedure that the researcher is testing for the first time will be treated at Aga Khan University, and its full responsibility will be borne by the research team/institute.

### Confidentiality

Your identity in this study will be treated as confidential. The results of the study will only be published for scientific purposes, and your name or any identifiable will not be included. There will be codes by which you will be identified, and the data will only be entered by the code. The questionnaire will be kept in lock and key. The results will not be shared with anyone. The study results will be shared in a scientific meeting and through publications.

### Termination of research study

You have full authority to participate in the study or refuse to participate in it. You also have the right to leave the study at any time during the study period. Your decision to leave the study will not affect the quality of treatment you receive at AKU.

### Available sources of information

If you have any questions regarding this study please call at this number: +922134864751

### Authorization

I have read or listen and understand this consent form, and I volunteer to participate in this research study. I understand that I will receive a copy of this form. I voluntarily choose to participate, but I understand that my consent does not take away any legal rights in the case of negligence or other legal faults of anyone who is involved in this study.

Name of participant: -----------------------

Signature or thumb impression of participant:-----------------

Date: ------------------------

Name of the Interviewer:------------------------

Signature of the Interviewer: ----------------------

Date: ---------------------

Name of the person obtaining consent: -------------------

Signature of person obtaining consent: -------------------

Date: ----------------------

Name of the witness

Signature of witness: -------------------

Date: ----------------------

Signature of PI/Co-PI: -------------------

Date: ---------------------

## Abbreviations

AKUH: Aga Khan University Hospital
ALND: Axillary lymph node dissection
SLNB: Sentinel lymph node biopsy
NCT: Neoadjuvant chemotherapy
ACOSOG: American College of Surgeons Oncology Group
FNR: False-negative rate
